# Special Issue “Molecular Advances and Perspectives of Lung Disease”

**DOI:** 10.3390/ijms26030946

**Published:** 2025-01-23

**Authors:** Jiacheng Jiang, Long Shuang Huang

**Affiliations:** 1 Shanghai Frontiers Science Center of Drug Target Identification and Delivery, School of Pharmaceutical Sciences, Shanghai Jiao Tong University, Shanghai 200240, China; cc0320@sjtu.edu.cn; 2 National Key Laboratory of Innovative Immunotherapy, Shanghai Jiao Tong University, Shanghai 200240, China

Respiratory diseases represent a significant global public health challenge, contributing to high mortality and morbidity rates worldwide. These conditions, including lung cancer, idiopathic pulmonary fibrosis, asthma, COVID-19, and tuberculosis, are characterized by complex pathophysiological mechanisms involving inflammatory responses, fibrosis, genetic diversity, microbiome dysbiosis, and disruptions in immune regulatory networks [1,2,3,4,5,6]. Inflammation and fibrosis are hallmark features of many respiratory diseases, while alterations in genetic diversity and the microbiome not only influence disease susceptibility but are also closely associated with disease progression and therapeutic outcomes [7,8,9]. As scientific research advances have increasingly demonstrated their critical roles in early diagnosis, personalized treatment, and prognostic evaluation, they have offered new approaches to address the clinical and societal challenges posed by lung diseases.

This Special Issue features nine carefully selected articles covering multiple cutting-edge areas of respiratory disease research. These include studies on genetic polymorphisms revealing disease susceptibility, multi-omics approaches elucidating key molecular networks, the discovery and validation of molecular biomarkers, and the identification of potential therapeutic targets and mechanisms. We hope that these studies will inspire new ideas for the academic and clinical communities, drive further progress in basic and applied research on respiratory diseases, and ultimately contribute to the global effort to address major health challenges.

The first topic is the recent progress of molecular markers and potential biomarkers in pulmonary diseases.

Lung squamous cell carcinoma (LSCC), a major subtype of non-small cell lung cancer (NSCLC), suffers from a lack of specific molecular markers, which limits early diagnosis and precision treatment [10]. By analyzing public databases and clinical samples, Fidelis et al. explored the potential biomarker role of *SCARA5* in LSCC [11]. Although *SCARA5* is widely recognized as a tumor suppressor gene in a variety of cancers, the results of the study showed that *SCARA5* expression level did not show a significant association with clinicopathological parameters such as tumor size and stage, as well as the overall survival rate of LSCC. Similarly, *SCARA5* expression levels did not significantly correlate with *THSD7A*-positive status, *FAK* expression levels, or *FGFR1* gene amplification. However, the study is the first report to analyze the clinicopathological significance of *SCARA5* in LSCC and its relationship with *THSD7A*, *FAK*, and *FGFR1*, providing preliminary data on the biological role of *SCARA5* in lung squamous cell carcinoma.

Idiopathic pulmonary fibrosis (IPF) is a progressive and incurable interstitial lung disease [12]. Treatment strategies primarily involve the use of antifibrotic agents, such as nintedanib and pirfenidone, to mitigate the progression of lung function decline [13,14]. Daisuke et al. found that the gene expression of PBMCs from IPF patients was significantly different from that of healthy controls, involving key processes such as the TGF-β signaling pathway, epithelial–mesenchymal transition (EMT), and lipid metabolism [15]. After drug treatment, differentially expressed genes in PBMCs showed different roles of pirfenidone (PFD) and nintedanib (NTD) in regulating fibrosis-related pathways. PFD treatment downregulated genes such as *COL1A1* (type I collagen) and *FERMT2*, hence attenuating fibroblast activity and collagen deposition. NTD inhibits TGF-β/Smad signaling by regulating lipid metabolism, which may reduce oxidative stress and fibrosis progression. Both drugs also ameliorate the course of pulmonary fibrosis by affecting coagulation pathways and integrin function, and it has been pointed out that the PBMC transcriptome reflects the systemic pathological process of IPF and can be used as a potential biomarker.

Tuberculosis (TB), a chronic infectious disease caused by *M. tuberculosis* (MTB), presents significant challenges in early diagnosis [6]. By integrating transcriptomics, proteomics, and metabolomics data, Anastasiia et al. analyzed the interactions between MTB and immune cells, such as alveolar macrophages, dendritic cells, B cells, and T cells, identifying key molecular markers [16]. These markers include cytokines (e.g., IL-1β, IL-6, IL-8, IFN-γ, TNF-α), matrix metalloproteinases (MMP-1, MMP-3, MMP-9), and their inhibitors (TIMP-1, TIMP-2, etc.). The study highlights that increased IFN-γ secretion, a higher proportion of CD69+ T cells, and the expression of CD137 and CD27 are crucial indicators for the early detection and prognosis of tuberculosis infection. Additionally, IL-1β levels can predict disease severity, while IL-6, VEGF, and serum amyloid proteins are closely linked to treatment outcomes. IL-8 and IL-1β may influence the progression of infection by modulating T cell and monocyte activity. These molecular markers not only aid in distinguishing different stages of tuberculosis but also help evaluate treatment efficacy and provide potential targets for drug development.

The second topic is the latest results on the role of genetic diversity and polymorphisms in pulmonary diseases.

Verónica et al. investigated the relationship between polymorphisms in inflammasome-related genes (e.g., *NLRP3*, *NLRC4*, *NLRP1*, *CARD8*, *IL1B*, *CASP1*, *IL18*) and the severity of COVID-19 [17]. Despite the known role of inflammasome hyperactivation in severe cases, the findings revealed no significant association between these gene polymorphisms and disease progression. Additionally, genetic differences between COVID-19 patients and healthy controls were not found. This suggests that the involvement of the inflammasome in severe COVID-19 may be due to changes in transcription or translation rather than genetic variations. These findings provide new insights into the role of inflammasomes in COVID-19 and underscore the need for further research into non-genetic regulatory mechanisms.

Another study looked at how vitamin D levels and changes in the vitamin D receptor (*VDR*) gene affect the risk of asthma in people from Eastern Europe (Latvia and Lithuania) and East Asia (Taiwan and Mongolia), comparing the results from different latitudes. Natalia et al. found no association of *FokI* (rs2228570) or *ApaI* (rs7975232) with asthma in the Baltic region, while *TaqI* (rs731236) demonstrated varying effects: the rare allele C increased asthma risk in Latvia but appeared protective in Lithuania [18]. Additionally, vitamin D levels were significantly lower in Lithuanian asthma patients compared to controls, a trend not observed in Latvia. Computational analysis identified that SNPs like rs731236 and rs1544410 affect transcription factor binding sites and DNA secondary structures, potentially influencing *VDR* gene expression and asthma risk.

The third topic is related to the progress of inflammation, immune regulation, and mechanisms in pulmonary diseases.

Pulmonary hypertension (PH) is a common complication of interstitial lung diseases (ILDs) and is associated with poor prognosis [19]. Tadasu et al. found that pulmonary vascular remodeling and media thickening were significantly attenuated in *Dpp4* knockout (*Dpp4*KO) mice [20]. In vitro, *DPP4*-siRNA treatment suppressed the TGFβ-induced proliferation of human pulmonary artery smooth muscle cells (hPASMCs). Transcriptome analysis revealed that TGFβ upregulated Notch3, PI3K-Akt, and NFκB signaling pathways related to hPASMC proliferation, which were reversed by *DPP4*-siRNA, including the downregulation of TGFBR1 and Notch3. The study underscores *DPP4*’s therapeutic potential in PH-ILD.

Chronic obstructive pulmonary disease (COPD) is a progressive lung disease characterized by airflow limitation and chronic inflammation [21]. Through RNA sequencing, Takuro et al. analyzed the transcriptomic profiles of peripheral blood mononuclear cells (PBMCs) from COPD patients and healthy controls, identifying 119 differentially expressed genes (DEGs) [22]. These genes were associated with airway remodeling, inflammation, and vascular abnormalities. The upregulation of immune-related genes like *BLNK* and *IL1R1* suggests PBMC involvement in airway inflammation, while *TFPI* and *PROS1* may protect the pulmonary vasculature via anticoagulant effects. KEGG analysis revealed a significant upregulation of the “hematopoietic cell lineage” pathway in non-emphysematous COPD, indicating an enhanced type 2 immune response. Genes such as *XCL1* and *PRKCZ* may be involved in lymphocyte and eosinophil migration. The upregulation of genes related to keratan sulfate synthesis (e.g., *ST3GAL3*, *B3GNT7*) may protect distal airways, while the upregulation of the Hedgehog signaling pathway could contribute to airway lesion formation. Additionally, *PAK4*, *COL6A1*, and *HSPG2* upregulation is linked to smooth muscle contraction, collagen deposition, and distal airway remodeling.

By integrating multiomics data, Beatriz et al. revealed how host genes and microbiomes communicate to regulate immune homeostasis and metabolic networks [23]. The oral–gut–lung microbiome is intricately interconnected through the lymphatic and circulatory systems, playing critical roles in immune homeostasis, inflammation regulation, and tumorigenesis. The oral microbiome influences gut and lung health through saliva and aerosols, with its dysbiosis linked to periodontal disease and systemic inflammation. Diet and lifestyle shape the gut microbiome, which is closely associated with inflammatory bowel diseases and cancer, with specific bacteria like Fusobacterium nucleatum playing pivotal roles in cancer progression. The lung microbiome undergoes significant changes in chronic respiratory diseases like COPD, where its imbalance correlates with worsening inflammation and disease progression. Cross-organ communication among microbiomes occurs via metabolites and immune effects, with dysbiosis potentially triggering systemic inflammation, disrupting immune balance, and elevating cancer risk. Host immunity interacts with microbiomes through MHC molecules and PRRs (e.g., TLRs), influencing inflammatory responses and gene expression. Microbial metabolites, such as short-chain fatty acids, regulate immune reactions, while dysbiosis can promote cancer immune evasion. Microbiome-targeted immunotherapies, such as immune checkpoint inhibitors, are emerging as promising strategies for cancer treatment.

PANoptosis integrates three programmed cell death pathways—apoptosis, pyroptosis, and necroptosis [24]. Shiyi et al. reviewed the critical role of PANoptosis in infections, inflammation, and tissue damage, significantly impacting the progression of lung diseases [25]. Pathogens activate immune responses, triggering cell death, which exacerbates inflammation. For example, influenza virus (IAV) and SARS-CoV-2 induce PANoptosis by activating immune receptors, disrupting the immune system and the lung microenvironment. Bacteria, fungi, and parasites can also trigger acute respiratory distress syndrome (ARDS). In asthma, allergens lead to immune responses that cause airway constriction and excessive mucus production, while NLRP3-dependent pyroptosis and MLKL-mediated necroptosis worsen the condition. In idiopathic pulmonary fibrosis (IPF) and chronic obstructive pulmonary disease (COPD), inflammasome activation and cell death drive lung fibrosis and airway inflammation. In cancer, PANoptosis may enhance immune cell activity, potentially helping to suppress tumor progression. Overall, the PANoptosome plays a key pathological role in various lung diseases by regulating cell death and inflammation responses.

Overall, this Special Issue systematically showcases the cutting-edge progress in molecular biomarkers and mechanism research in respiratory diseases, emphasizing the important value of interdisciplinary integration in elucidating the complex mechanisms of these diseases. These studies not only deepen our understanding of respiratory diseases but also provide strong support for personalized treatment in clinical practice.

Looking ahead, research on respiratory diseases will further develop in depth, focusing on both basic research and clinical validation. It is recommended to explore emerging mechanisms such as *SCARA5*, CD26/DPP4, and PANoptosis in greater detail and extend their application to other diseases. At the same time, the clinical validation of biomarkers will accelerate their practical use in early diagnosis and treatment, particularly in COVID-19 and tuberculosis. Furthermore, with the continuous advancement of single-cell genomics, spatial transcriptomics, and artificial intelligence, future research will make further breakthroughs in understanding the dynamic impact of the microenvironment on disease progression, providing stronger technical support for the development of precision medicine. We look forward to closer collaboration between basic and clinical research, promoting multi-omics data integration, and opening new prospects for the early diagnosis and personalized treatment of respiratory diseases.
